# A study of pulmonary function in end-stage renal disease patients on hemodialysis: a cross-sectional study

**DOI:** 10.1590/1516-3180.2017.0179150817

**Published:** 2017-09-28

**Authors:** Ashima Sharma, Ashok Sharma, Sushila Gahlot, Pawan Kumar Prasher

**Affiliations:** I MD. Senior Resident, Department of Physiology, Indira Gandhi Medical College, Shimla, India.; II MD. Professor, Department of Medicine, Indira Gandhi Medical College, Shimla, India.; III MD. Professor, Department of Physiology, Gian Sagar Medical College & Hospital, Ramnagar, Patiala. India; IV MD, DM. Professor, Department of Medicine, Gian Sagar Medical College & Hospital, Ramnagar, Patiala. India

**Keywords:** Kidney diseases, Respiratory function tests, Renal dialysis

## Abstract

**BACKGROUND:**

The aim here was to study acute effects of hemodialysis among end-stage renal disease (ESRD) patients.

**DESIGN AND SETTING:**

Prospective study in tertiary-level care center.

**METHODS:**

Fifty ESRD patients undergoing hemodialysis were studied. Spirometric pulmonary function tests were performed before and after four-hour hemodialysis sessions.

**RESULTS:**

The patients’ average age was 45.8 ± 10.0 years; 64% were males and 64% had normal body mass index. Anemia (94%) and hypoalbuminemia (72%) were common. Diabetes mellitus (68%), hypertension (34%) and coronary artery disease (18%) were major comorbidities. Forty-five patients (90%) had been on hemodialysis for six months to three years. The patients’ pre-dialysis mean forced vital capacity (FVC) and forced expiratory volume in 1 second (FEV1) were below normal: 45.8 ± 24.9% and 43.5 ± 25.9% of predicted, respectively. After hemodialysis, these increased significantly, to 51.1 ± 23.4% and 49.3 ± 25.5% of predicted, respectively (P < 0.01). The increase in mean FEV1/FVC, from 97.8 ± 20.8% to 99.3 ± 20.1% of predicted, was not significant (P > 0.05). The pre-dialysis mean forced expiratory flow 25-75% was 50.1 ± 31% and increased significantly, to 56.3 ± 31.6% of predicted (P < 0.05). The mean peak expiratory flow was below normal (43.8 ± 30.7%) and increased significantly, to 49.1 ± 29.9% of predicted (P < 0.05). Males and females showed similar directions of change after hemodialysis.

**CONCLUSIONS:**

Pulmonary function abnormalities are common among ESRD patients. Comparison of pre and post-hemodialysis parameters showed significant improvements, but normal predicted values were still not achieved.

## INTRODUCTION

Chronic kidney disease (CKD) is a worldwide public health problem and comprises the presence of sustained and irreversible abnormality of renal functions and loss of the kidneys’ ability to maintain homeostasis. CKD results from different causes of renal injury and can lead to progressive loss of renal function. It may reach end-stage renal disease (ESRD) after a variable period of time following the initiating injury. ESRD is the situation when kidney function is insufficient to sustain life and there is then a need for hemodialysis (HD), peritoneal dialysis (PD) or kidney transplantation, to substitute for native kidney function.^1^ ESRD presents not only as progressive and irreversible loss of excretory function of the kidneys but also as a complex syndrome with altered metabolic and endocrine functions. It has effects on almost all body systems, including pulmonary function.^2^

The relationship between the lungs and the kidneys is clinically important for both health and disease.^3^ Kidney failure directly and indirectly impacts the mechanical function and ventilation of the lungs, and treatment with drugs and HD are responsible for part of this effect.^4^ Patients with ESRD require dialysis in the form of HD or peritoneal dialysis for survival, because these can partially replace the impaired kidney function, reverse the uremic symptoms and preserve patients with ESRD, while they await a definitive solution through kidney transplantation, if possible.^5^ Pulmonary function tests have been compared among individuals on HD and peritoneal dialysis and among kidney transplant recipients, and it was found that pulmonary restrictive defect was the most common dysfunction in all these groups.^6^

Further spirometric changes were studied by Lang et al. before and after HD using different dialyzer membranes, and they found that there was no significant difference between pre-HD and post-HD vital capacity.^7^ On the other hand, in a similar study on the acute effects of HD, Rahgoshai et al. demonstrated that pulmonary function, and especially forced vital capacity (FVC), improved after a HD session; while no significant improvements in forced expiratory volume in 1 second (FEV1), FVC or FEV1/FVC ratio were observed.^8^

Dialysis may have beneficial effects at least in the initial stages of some respiratory disorders among CKD patients without primary lung disease. It may lead to improvement of respiratory symptoms and even pulmonary function test values.^9^ However, the immune response resulting from contact between blood and bioincompatible dialysis filters may cause complement activation, which can have a deteriorating effect on the respiratory system and can even cause respiratory distress.^10^

HD-related hypoxemia is another issue among ESRD patients. HD can reduce pulmonary edema around the small airways, and this may lead to dilation of the small airways (decreased closing capacity). It also gives rise to improved basal ventilation and perfusion.^11^

The effects of HD on patients with CKD relate mainly to changes to the volume of body fluid, thus leading to reduction of the amount of water in the lungs following dialysis. Hence, HD improves respiratory status but it may cause pulmonary complications as well, due to various pulmonary injuries of multifactorial origin. Moreover, the malnutrition and degenerative alterations that can occur in CKD patients persist, thereby worsening muscle loss and predisposing these patients towards fatigue, with increases in respiratory rate and work.^12^

## METHODS

**Design:** Our study was prospective and was conducted over a one-year period from November 2011 to December 2012, in a tertiary-level care center. It was an observation study. It was conducted on 50 ESRD patients undergoing HD. The study was approved by our institution’s research ethics committee.

**Inclusion criteria:** Ambulatory, clinically stable patients in the age group of 18-60 years, who had been undergoing HD for more than three months, were included in the study.

**Exclusion criteria:** Patients with histories of smoking (current or previous), acute infection, acute renal failure, chronic lung disease, tuberculosis, skeletal muscle abnormality, decompensated heart failure, arrhythmias or liver cirrhosis, and patients in severe respiratory distress or who were unable to undergo spirometry, as assessed by the clinician administering the treatment, were excluded from the study.

The patients were given explanations regarding the purpose of the study and written informed consent was obtained from them. Individuals’ data were recorded on an assessment form. The glomerular filtration rate (GFR) was estimated by using the empirical formula for creatinine clearance (Cockcroft-Gault equation).

HD was performed using the Fresenius Medical Care 4008-S, a German machine. While the patients’ blood flow range was variable from 300 to 350 ml/min, the dialysate flow was constant (500 ml/min). Dialysis was done using a biocompatible membrane and bicarbonate buffer. Intra-dialysis ultrafiltration was based on the patients’ condition and on their weight gain during the interdialytic period.

The pulmonary function tests were performed using a computerized spirometer (“Medicaid Spirometer”). This automatically corrected all gas volumes to body temperature and pressure, saturated (BTPS), i.e. a set of conditions at body temperature, with ambient pressure and with saturation with water vapor. Spirograms (flow volume and volume-time graphs) were produced, along with numerical data and the predicted percentage values for the spirometric parameters. Spirometric variables were recorded 15 minutes before and after the first HD session of the week.

The data obtained were analyzed statistically with the aid of the Statistical Package for the Social Sciences (SPSS) for personal computer, version 11.0, and paired t tests were used for comparative analyses. P < 0.05 was taken to be significant and P < 0.01 was taken to be highly significant.

## RESULTS

This study was conducted from December 2011 to December 2012. A total of 50 patients met our inclusion criteria and were included in the study. The mean age of the study patients was 45.8 ± 10.0 years (range 24 to 60 years). The majority (62%) of the patients were below fifty years of age. Males formed the predominant group among the patients (64%) and 72% of the patients were from a rural background. The majority (64%) of the patients had a normal body mass index (BMI). BMI did not differ statistically according to gender (Table 1).

**Table 1:** Body mass index status of study patients (in kg/m^2^)

Body mass index (kg/m^**2**^ )	No. of patients (n)	(%)
Underweight (< 18.5)	10	20
Normal (18.5-24.99)	32	64
Overweight (25-29.99)	8	16
Obese (> 30)	0	0

On investigation, 94% of the patients were anemic (hemoglobin < 12 g%). The hemoglobin values ranged from 5.0 to 12.5 g/dl (mean ± standard deviation, SD: 9.5 ± 1.6) among all the patients. For males, the hemoglobin range was 7.5-12.5 g/dl (10.2 ± 1.3), while for females, the values were 5.0-11.0 g/dl (8.3 ± 1.5).

The serum albumin levels ranged from 2.4 to 4.2 gm/dl. The mean serum albumin concentration was 3.28 ± 0.48 and 72% of the patients had hypoalbuminemia. Pre-dialysis serum urea levels ranged from 92.0 to 278.0 mg/dl (163.1 ± 39.1 mg/dl), serum creatinine 4.6-19.7 mg/dl (9.9 ± 3.3 mg/dL) and estimated GFR 3.0-19.8 ml/min/1.73 m^2^ (7.8 ± 3.4 ml/min/1.73 m^2^). There were no statistical differences in baseline urea and creatinine levels according to gender, but estimated GFR was significantly higher in males (8.8 ± 3.5 ml/min/1.73 m^2^) than in females (6.0 ± 2.3 ml/ min/1.73 m^2^).

Forty-five patients (90%) had been on HD for six months to three years and only 10% had been on HD for less than six months. The proportions were identical for males and females (Table 2). The majority (58%) of the study patients (62% of the males and 50% of the females) were undergoing HD twice a week, while 30% of all the patients (31% of the males and 28% of the females) were undergoing HD three times a week. Eight percent of the study patients were on hemodialysis once a week and another four percent once every two weeks (Table 3). 68% of the patients were diabetic before they were diagnosed as having CKD, while 34% were known to be hypertensive and 18% had coronary artery disease.

**Table 2:** Duration of hemodialysis among study patients

Duration of hemodialysis	Males (n = 32)	Females (n = 18)	Total (n = 50)
< Six months	3 (9.4%)	2 (11.1%)	5 (10.0%)
Six months to one year	12 (37.5%)	7 (38.9%)	19 (38.0%)
> One year to three years	17 (53.1%)	9 (50.0%)	26 (52.0%)

**Table 3:** Frequency of hemodialysis among study patients

Frequency of hemodialysis	Total (n = 50)	%
Three times a week	15	30
Twice a week	29	58
Once a week	4	8
Once every two weeks	2	4

The percentages of the predicted spirometric parameters (% pred) and changes to spirometric parameters among our study patients after hemodialysis are presented in Table 4 and Table 5.

**Table 4:** Percentage of predicted spirometric parameters among the study patients (before and after hemodialysis [HD]) (n = 50)

Spirometric parameters	Before HD (mean ± SD)	After HD (mean ± SD)	Paired differences				
			Mean	SD	SEM	95% CI	P-value
FVC	45.8 ± 24.9	51.1 ± 23.4	5.3	8.6	1.2	2.8-7.7	< 0.001
FEV1	43.5 ± 25.9	49.3 ± 25.5	5.8	9.4	1.3	3.2-8.6	< 0.001
FEV1/FVC (%)	97.8 ± 20.8	99.3 ± 20.1	1.5	18.0	2.6	-3.6-6.6	0.561
FEF 25-75%	50.1 ± 31.3	56.3 ± 31.6	6.2	20.9	3.0	0.2-12.1	0.043
PEFR	43.8 ± 30.7	49.1 ± 29.9	5.3	13.1	1.9	1.6-9.1	0.006

SD = standard deviation; SEM = standard error of the mean; CI = confidence interval; P-value from paired t-test (two-tailed); FVC = forced vital capacity; FEV1 = forced expiratory volume in 1 second; FEV1/FVC (%) = ratio between FEV1 and FVC x 100; FEF 25-75% = forced expiratory flow in 25-75% of FVC; PEFR = peak expiratory flow rate. Note: The mean FEV1/FVC (%) of the study patients was 97.8 ± 20.8% of the predicted value.

**Table 5:** Change in spirometric parameters among patients after hemodialysis

Spirometric parameter	Male (n = 32)		Female (n = 18)		P-value
	Increase		Increase		
	N	%	N	%	
FVC	25	78.1	15	83.3	0.941
FEV1	24	75.0	14	77.8	0.901
FEV1/FVC ratio	21	65.6	10	55.6	0.689
FEF 25-75%	17	53.1	13	72.2	0.307
PEFR	19	59.4	11	61.1	0.856

FVC = forced vital capacity; FEV1 = forced expiratory volume in 1 second; FEF = forced expiratory flow; PEFR = peak expiratory flow rate.

**FVC:** The mean FVC of the study patients was 45.8 ± 24.9% pred, i.e. well below the normal predicted values for pulmonary function (normal is more than 80% of the predicted values), determined through spirometry. After HD, the mean FVC increased to 51.1 ± 23.4% pred, and this increase was statistically highly significant (P < 0.01) (Table 4).

**FEV1:** The mean FEV1 of the study patients was 43.5 ± 25.9% pred, which was also well below the normal predicted values (normal is more than 80% of the predicted values). After HD, the mean FEV1 increased to 49.3 ± 25.5% pred, and this increase was statistically highly significant (P < 0.01) (Table 4**)**.

**FEV1/FVC%:** The mean FEV1/FVC% of the study patients was 97.8 ± 20.8% pred. After HD, the mean FEV1/FVC% increased to 99.3 ± 20.1% pred, but this increase was not statistically significant (P > 0.05) (Table 4**)**.

Forced expiratory flow **(FEF) 25-75%:** The mean FEF 25-75% of the study patients was 50.1 ± 31% pred. After HD, the mean FEF 25-75% increased to 56.3 ± 31.6% pred, and this increase was statistically significant (P < 0.05) (Table 4**)**.

Peak expiratory flow rate **(PEFR):** The mean PEFR of the study patients was 43.8 ± 30.7% pred, i.e. well below the normal range (normal is more than 80% of the predicted values). After HD, the mean PEFR increased to 49.1 ± 29.9% pred, and this increase was statistically significant (P < 0.05) (Table 4).

The overall analysis on pulmonary function in our study revealed that the majority of the patients (82%) had a normal FEV1/FVC ratio (> 70%) and low percentages of the predicted FVC value (< 80% pred), which was indicative of restrictive pulmonary disorder. Moreover, 6% had an FEV1/FVC ratio less than 70%, which indicates obstructive respiratory disorder. However, because these patients also had low FVC, they could be included in the category of mixed respiratory disorder. Only 12% of the study patients had pulmonary function in the normal range.

There were no statistically significant differences in any of the spirometric parameters after HD when compared on the basis of gender. Males and females showed similar directions of change after HD (Table 5).

## DISCUSSION

The mean age of our study patients was 45.8 ± 10.0 years and 62% of them were < 50 years, thus suggesting that CKD had emerged as an early complication of various disorders. The preponderance of males (64%) may have reflected either greater prevalence of CKD among males or, alternatively, poor availability of costly HD treatment for female patients, due to various sociocultural and economic constraints. The mean BMI of the study group was 21.6 ± 3.0 kg/m^2^ and 20% had BMI < 18.5 kg/m^2^, but the majority (64%) of the patients had normal BMI. This was despite chronic illness, but the patients’ obvious water retention and edema may have led to their normal BMI.

Anemia (94%) and hypoalbuminemia (72%) were very prevalent. This may have been due to higher levels of renal dysfunction and poor nutritional status, reflecting both an inflammatory state and poor nutritional status among the patients. The high prevalence of these conditions may also have been due to a hypercatabolic state in CKD, caused by accumulation of proinflammatory cytokines and a combination of factors like uremic toxicity, insulin resistance, and amino acid losses^13^ during the dialysis procedure, rather than mere lack of a high protein diet.^14^

### FVC (forced vital capacity)

The mean FVC of the study group was 45.8 ± 24.9% pred, i.e. well below the normal predicted values for pulmonary function. After HD, the mean FVC increased to 51.1 ± 23.4% pred (P < 0.001). Our findings regarding FVC are in agreement with Mehmood.^7^^,^^15^^,^^16^^,^^17^ Decreased FVC, restrictive pattern and reduced airflows have been observed through spirometry, in studies by several authors. Chronic subclinical pulmonary edema due to increased capillary permeability and hypoalbuminemia was considered to be the cause for the decreased FVC.^11^^,^^18^

### FEV1

The mean FEV1 of our study patients was 43.5 ± 25.9% pred. which was also well below the normal predicted values (normal is more than 80% of the predicted values). It showed a statistically significant increase after HD, to 49 ± 25.5% pred (P < 0.01). However, these low FEV1 values were associated with a normal FEV1/FVC ratio in most of our patients, which suggested that the large airways were not affected in situations of chronic renal failure and that the reduction of FEV1 was primarily due to reduction in FVC, as in restrictive pulmonary disease. Reduced FEV1 as observed in our study patients has also been reported by Maehmood et al. and Nascimento et al.^15^^,^^16^ Inflammation and malnutrition have been found to present significant relationships with reduced pulmonary parameters.^19^

### FEV1/FVC percentage ratio

Most of the study patients (82%) had a normal FEV1/FVC ratio (> 70%) and reduced FVC (i.e. < 80% of the predicted values), which was indicative of restrictive pulmonary disorder, while 6% had an FEV1/FVC ratio of less than 70%, thus pointing towards obstructive respiratory disorder. However, the latter patients also presented decreased FVC and could be included in the category of mixed respiratory disorder. Only 12% of the study patients had pulmonary function within the normal range. The mean FEV1/FVC ratio of our patients was 97.8 ± 20.8% pred, and this increased to 99.3 ± 20.1% pred) after HD, but this increase was not statistically significant (P > 0.05). In contrast to FEV1 and FVC, there were no significant changes overall or among the subgroups of the study patients regarding the FEV1/FVC ratio after HD, because there was corresponding increase in both parameters (FVC and FEV1). No significant change in this ratio was also observed by Navari et al.^4^

### FEF 25-75% (forced expiratory flow over the middle part of FVC)

FEF 25-75% is measured from a segment of the FVC that includes flow from medium and small airways. Decreased flows are common in the early stages of obstructive disease. In the presence of borderline values for FEV1/FVC, a low FEF 25-75% confirms airway obstruction and sometime signals decreased cross-sectional area in the small airways. The mean FEF 25-75% among our study patients was 50.1 ± 31.3% pred and, after HD, it increased to 56.3 ± 31.6% pred, which was a statistically significant increase (P < 0.05). Rakovaca et al. found decreased values for FEF 25-75% among ESRD patients.^20^ Mahmood et al. also observed decreased values that were indicative of small airway disease, in comparison with their controls.^15^

The improvement in FEF 25-75% that was achieved through HD among our study patients showed that there was a situation of reversible obstruction, comprising removal of excess fluid from the lungs that had been compressing the small airways. However, chronic subclinical pulmonary edema leading to peribronchial fibrosis may also contribute towards the persistent abnormalities in the small airways that are reflected in reduced FEF 25-75% values.

### PEFR (peak expiratory flow rate)

Among our study patients, the mean PEFR was 43.8 ± 30.7% pred, i.e. below the normal range. After HD, the PEFR increased to 49.1 ± 29.9% pred, and this increase was statistically significant (P < 0.05). Mahmood et al. reported that the values among CKD patients on HD were lower than those of normal subjects.^15^ Reduced PEFR before and even during HD sessions was observed by Davenport, who attributed this to activation of the complement system for neutrophils, monocytes and platelets following blood membrane interaction, thereby resulting in appreciable airway constriction.^21^ In contrast to the findings from our study, normal PEFR values were observed by Lang et al.^7^

## CONCLUSION

Pulmonary function abnormalities were common among our study patients, but were significantly ameliorated after HD. The majority of our patients had restrictive and mixed respiratory disorders. In the pulmonary function tests on our ESRD patients, spirometric parameters like FVC, FEV1 and PEFR were less than the normal predicted values (i.e. < 80% of the predicted values) in the majority of these patients. In comparing the pre-HD and post-HD spirometric parameters, there was significant improvement but normal predicted values were still not achieved.
